# ALIAmides and pediatric health: a structured narrative review

**DOI:** 10.3389/fphar.2026.1900560

**Published:** 2026-09-15

**Authors:** Alessandro Vittori, Ilaria Mascilini, Massimiliano Valeriani, Marco Cascella, Giovanni Di Nardo, Arianna Di Stadio, Domenico Bove, Alessandro Ferretti, Laura Papetti, Elisa Francia

**Affiliations:** 1 Department of Anesthesia, Critical Care and Pain Medicine, ARCO, Ospedale Pediatrico Bambino Gesù IRCCS, Rome, Italy; 2 Developmental Neurology Unit, Bambino Gesù Children’s Hospital IRCCS, Rome, Italy; 3 Systems Medicine Department, Tor Vergata University of Rome, Rome, Italy; 4 Department of Health Science and Technology, Translational Pain Neuroscience and Precision Medicine, School of Medicine, CNAP, Aalborg University, Aalborg, Denmark; 5 Department of Medicine, Surgery and Dentistry, University of Salerno, Baronissi, Italy; 6 Pediatric Gastroenterology and Endoscopy Unit, Department of Pediatric Specialties, Santobono Pausilipon Children’s Hospital, Naples, Italy; 7 Department of Neurosciences, Mental Health and Sensory Organs (NESMOS), Sapienza University of Rome, Rome, Italy; 8 Life Sciences, Health and Health Professions Department, LINK University, Rome, Italy; 9 Neuropsychiatry, Psychotherapy and Rehabilitation “Centro Clinico FUSIS”, Caserta, Italy; 10 Pediatrics Unit, Neuroscience, Mental Health and Sense Organs (NESMOS) Department, Faculty of Medicine and Psychology, S. Andrea Hospital, Sapienza University of Rome, Rome, Italy

**Keywords:** adelmidrol, gastrointestinal and genitourinary disorders, micron-sized formulations, neurocognitive disorders, otorhinolaryngologic diseases, pain, palmitoylethanolamide, pediatric population

## Abstract

**Introduction:**

Although most children require medications only for occasional acute conditions, a significant proportion lives with chronic diseases that necessitate long-term, often multiple treatments, which negatively impact their health and quality of life, and may lead to adverse outcomes. Challenges related to prescribing and formulation further complicate pediatric treatment, underscoring the need for safe and effective innovative strategies. Autacoid Local Injury Antagonism amides (ALIAmides), particularly palmitoylethanolamide (PEA) and Adelmidrol, represent promising therapeutic options. This structured narrative review aims to collect and discuss all available literature on PEA and Adelmidrol use in pediatric patients.

**Methods:**

A search of PubMed, Scopus, and the Cochrane Library identified 17 studies involving a total of 2,530 patients, of whom 1,772 received PEA (oral or topical), and 54 received Adelmidrol. Following a transdiagnostic approach, results were organized by symptom domains rather than by specific diagnoses, to capture potential benefits across multiple conditions and facilitate comparison with previous studies. Data extraction included study design, population characteristics, intervention type and formulation, outcomes assessed, and main findings.

**Results:**

Beneficial effects on behavioral, cognitive, motor, and visceral functions, along with improvements in respiratory and dermatological disorders emerged from studies administering oral PEA, mostly in micron-sized formulations. Topical application of both PEA and Adelmidrol also demonstrated benefits on respiratory and skin conditions.

**Conclusion:**

Overall, the findings of this review suggest that PEA and Adelmidrol may represent promising, well-tolerated adjunctive strategies for managing pediatric symptoms across various clinical conditions, particularly those that are chronic or require polypharmacy.

## Graphical Abstract

**Figure:**
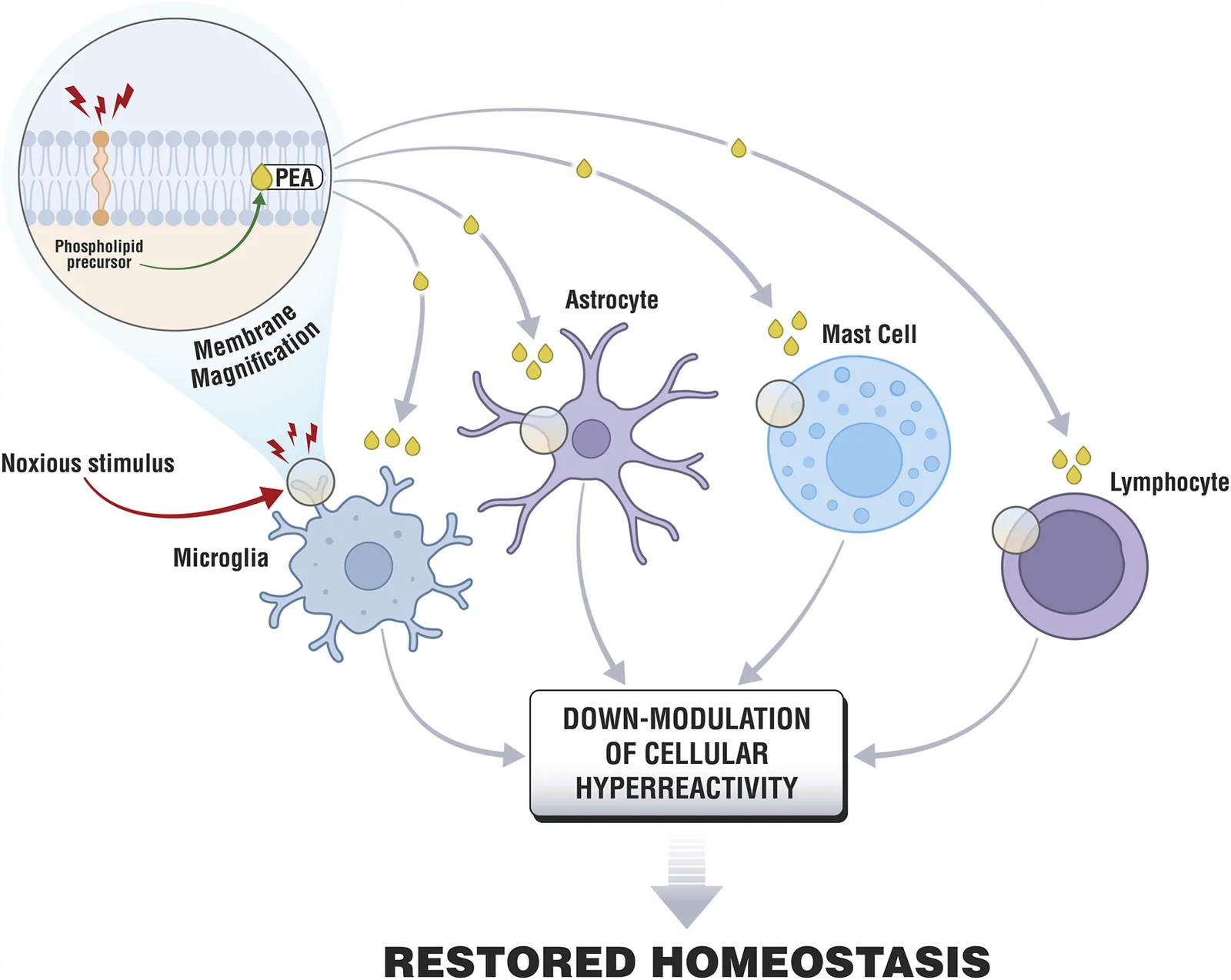

## 1 Introduction

The pediatric population typically requires limited medication use, mainly for occasional acute conditions. Yet a substantial proportion of children suffer from chronic disorders and often require long-term management involving multiple concomitant treatments, with significant impact on health, daily routines, emotional wellbeing, and family dynamics (Ewig et al., 2022; Mastorci et al., 2025). Polypharmacy has been frequently associated with adverse outcomes in children, including drug resistance, tolerance, and severe side effects (Juárez-Hernández and Carleton, 2022; Basu, 2024).

Infancy and childhood are periods of rapid growth, during which organs, physiological systems, and drug-metabolizing enzymes mature at different rates, resulting in heterogeneous pharmacokinetic and pharmacodynamic responses and variable susceptibility to adverse effects (Bereda, 2022). Limited ability to communicate medication-related adverse effects, inappropriate prescriptions, insufficient experience in pediatric pharmacotherapy, and the lack of age-appropriate formulations further complicate safe treatment, highlighting the need for innovative strategies to minimize adverse effects while optimizing therapeutic outcomes (Juárez-Hernández and Carleton, 2022).

In this context, ALIAmides–fatty acid amides named for their Autacoid Local Injury Antagonism (ALIA) mechanism of action–represent a promising therapeutic option. First identified by the Nobel Laureate Rita Levi Montalcini, the ALIA mechanism refers to the endogenous, on-demand production of protective lipid mediators from membrane phospholipid precursors in response to noxious stimuli, aimed at restoring cellular homeostasis (Aloe et al., 1993; Levi-Montalcini et al., 1996). Originally identified at the level of mast cells, the ALIA mechanism has subsequently been recognized as a broader self-protective and pro-homeostatic system, operating across multiple cell types, including immune cells, such as lymphocytes as well as non-neuronal cells, notably microglia (Chiurchiù et al., 2018; D’amico et al., 2020; Gugliandolo et al., 2020; Palenca et al., 2025) (Figure 1). Endogenous ALIAmides include the ethanolamides of palmitic, oleic, and stearic acids, namely, palmitoylethanolamide (PEA), oleoylethanolamide (OEA), and stearoylethanolamide (SEA).

**FIGURE 1:**
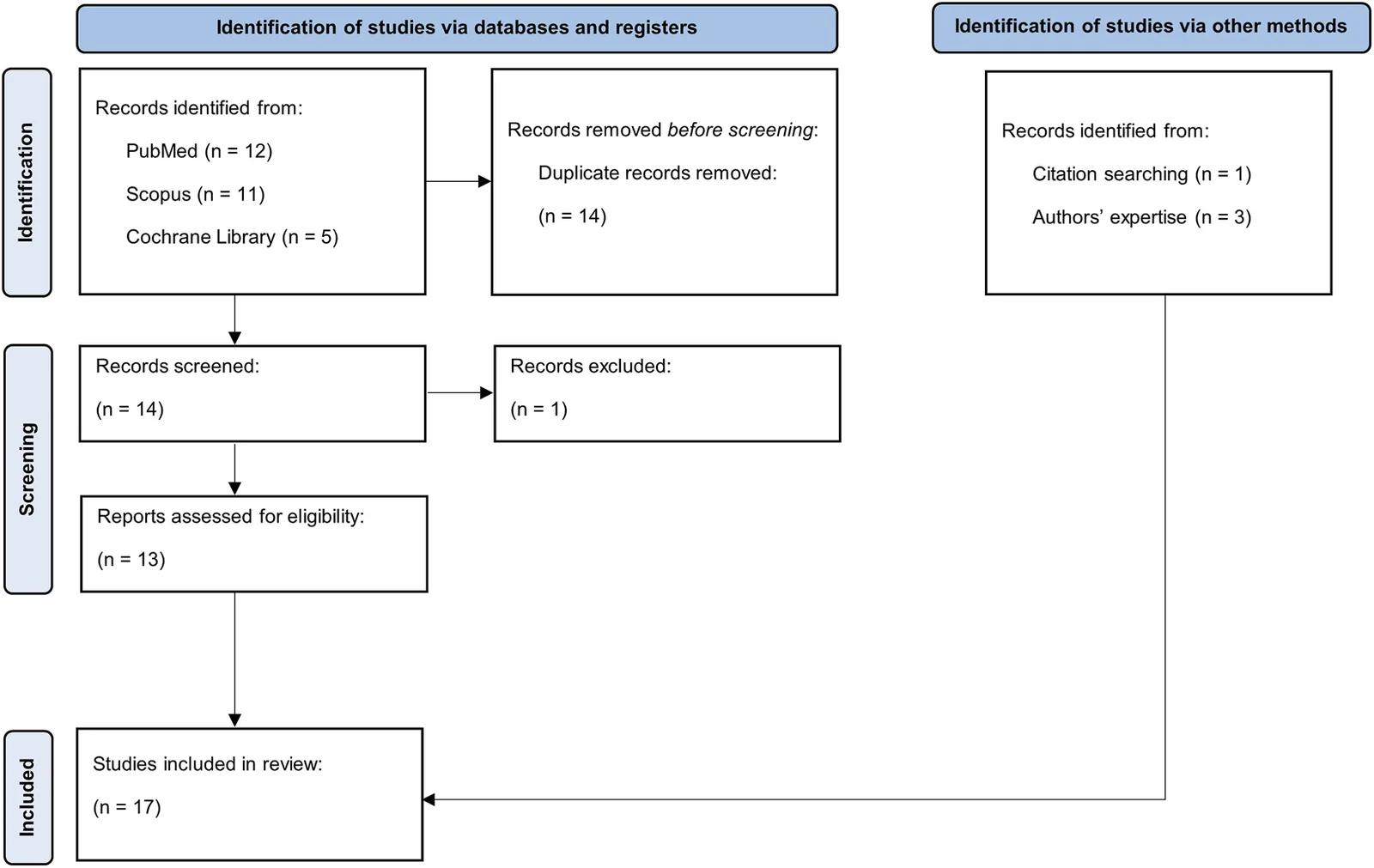
Flowchart of the literature search.

PEA has drawn particular attention for its anti-inflammatory, neuroprotective, immunomodulatory, and anti-hyperalgesic properties, mainly achieved by modulating mast cells and microglia (Nobili et al., 2024). Its protective effects were first noted in 1943, when Coburn and Moore observed that an egg-rich diet prevented recurrence of rheumatic fever in children (Coburn and Moore, 1943). Subsequent studies identified PEA as the active factor, present not only in egg yolk but also in various foods as well as body tissues and fluids, including amniotic fluid, umbilical blood vessels, and breast milk, highlighting its physiological relevance from the perinatal period onward (Gugliandolo et al., 2020; Petrosino and Schiano Moriello, 2020). PEA contributes to neonatal growth and feeding regulation, and its levels can be influenced by maternal diet, with potential long-term effects on metabolism, neurobehavior, and susceptibility to inflammation-related disorders (Ramírez-López et al., 2016b; Ramírez-López et al., 2016a; Bruun et al., 2018; Gaitán et al., 2019). Nevertheless, under prolonged stress–especially during early development–endogenous levels of PEA may be insufficient, supporting exogenous supplementation to restore homeostasis (Clayton et al., 2021; Di Stefano et al., 2025). Experimental models indeed show that early PEA administration mitigates neurodevelopmental damage after hypoxia or perinatal asphyxia, reduces neuroinflammation, preserves memory and locomotor functions, and improves neurobehavioral outcomes, indicating its potential to support brain development and resilience to injury (Herrera et al., 2018; Herrera et al., 2022; Holubiec et al., 2018; Portavella et al., 2018).

Alongside PEA, the semisynthetic ALIAmide Adelmidrol (the diethanolamide of azelaic acid) has emerged as a promising compound. Its amphipathic nature facilitates solubility in both aqueous and lipid environments, enhancing clinical versatility (i.e., topical and intracavitary administration). Adelmidrol acts as a preventive antioxidant through its radical-scavenging activity, thereby protecting hyaluronic acid from oxidative degradation and reducing mast cell degranulation (D’amico et al., 2020; Cavallaro et al., 2024).

Formulations of both PEA and Adelmidrol have demonstrated efficacy and excellent safety profiles in adult clinical settings (Indraccolo et al., 2013; Clayton et al., 2021; Scavone, 2021; Teobaldi et al., 2022; Kurti et al., 2023; Nobili et al., 2024; Bortoletto et al., 2025), providing a rationale for pediatric application, particularly in chronic conditions. Given their potential impact on children’s health and quality of life, ALIAmides warrant careful consideration. This review aims to comprehensively collect and discuss available data on the efficacy and safety of PEA and Adelmidrol across pediatric clinical disorders.

## 2 Materials and methods

The review was conducted as a structured narrative review, adhering to the Scale for Assessment of Narrative Review Articles (SANRA) guidelines (Baethge et al., 2019). To further enhance methodological rigor, systematic elements inspired by the Preferred Reporting Items for Systematic Reviews and Meta-Analyses (PRISMA) were also incorporated.

To ensure comprehensiveness, multiple sources of evidence were integrated. The PubMed, Scopus and Cochrane Library databases were systematically searched up to March 2026 using appropriate keywords and Boolean operators: (Palmitoylethanolamide OR N-Palmitoylethanolamine OR Adelmidrol) AND (children OR pediatric). A supplementary search was performed by screening the reference lists of the selected papers to identify additional relevant studies. Further literature was identified based on the authors’ expertise. No restrictions were applied regarding publication date or language.

Studies were considered eligible if they met the following criteria: (i) included pediatric populations; (ii) addressed any clinical condition; (iii) investigated treatment with PEA or Adelmidrol, either alone or in combination with other therapies; (iv) adopted study designs such as case reports, observational studies and randomized controlled trials (RCTs); and (iv) reported outcomes assessed through appropriate clinical measures. Narrative or systematic reviews, meta-analyses, and book chapters were excluded.

Following a transdiagnostic approach previously described by Bortoletto and colleagues (Bortoletto et al., 2025), results were organized by symptom domains rather than by specific diagnoses, to capture potential cross-condition effects and facilitate comparison with prior literature.

Data extraction included study design, population characteristics, intervention type and formulation, outcomes assessed, and main findings. To enhance critical appraisal, study designs were classified, and levels of evidence were assigned in accordance with the Oxford Centre for Evidence-Based Medicine (CEBM) guidelines.

Methodological quality and risk of bias were assessed using validated tools. Randomized controlled trials were evaluated using the Cochrane Risk of Bias 2 (RoB 2) tool (Sterne et al., 2019). Non-randomized studies were assessed using the Risk Of Bias In Non-randomized Studies–of Interventions (ROBINS-I) framework (Sterne et al., 2016), with domains interpreted according to the specific characteristics of each study, particularly for uncontrolled designs. Risk-of-bias traffic light plots were generated using the Robvis visualization tool (McGuinness and Higgins, 2021). Case reports were appraised using the Joanna Briggs Institute (JBI) Critical Appraisal Checklist for Case Reports (Joanna Briggs Institute, 2026).

## 3 Results

### 3.1 Study selection and description

A total of 32 publications were identified from the initial search. After removing duplicates, 18 articles were retrieved. Following the full-text evaluation, one additional paper was excluded because it was not related to a clinical trial. Consequently, 17 studies were considered (Figure 2). Among these, three case reports (Antonucci et al., 2015; Bertolino et al., 2017), one observational study with preliminary findings reported as a letter (Bove, 2024), and one RCT (Khalaj et al., 2018) investigated the effects of PEA supplementation in children diagnosed with autism spectrum disorder (ASD). One case report described the outcomes of a child with traumatic brain injury (TBI) managed with PEA (De Palma et al., 2018), while a multicenter RCT and a pilot study evaluated PEA in pediatric populations with irritable bowel syndrome (IBS) (Di Nardo et al., 2024b) and migraine without aura (Papetti et al., 2020), respectively. PEA was also investigated in one case report describing a pediatric patient with multiple neurodevelopmental disorders and severe headaches (Turrini et al., 2025) and in one observational study investigating pediatric pain related to limb-lengthening surgery (Kotzeva et al., 2025), both of which were conference abstracts published in a peer-reviewed journal. Five studies (Rasková et al., 1974; Plesník et al., 1977; Nigro et al., 2014; Ameli and Ciprandi, 2019; Della Volpe et al., 2025) explored the effects of PEA or Adelmidrol on respiratory infections, and three others examined their use in various dermatological conditions (Del Rosso, 2007; Pulvirenti et al., 2007; Eberlein et al., 2008). A detailed overview of the included studies is provided in Table 1, organized hierarchically by their level of evidence and in reverse chronological order, summarizing study design, level of evidence, patient numbers, intervention dose and duration, formulation, and main findings.

**FIGURE 2:**
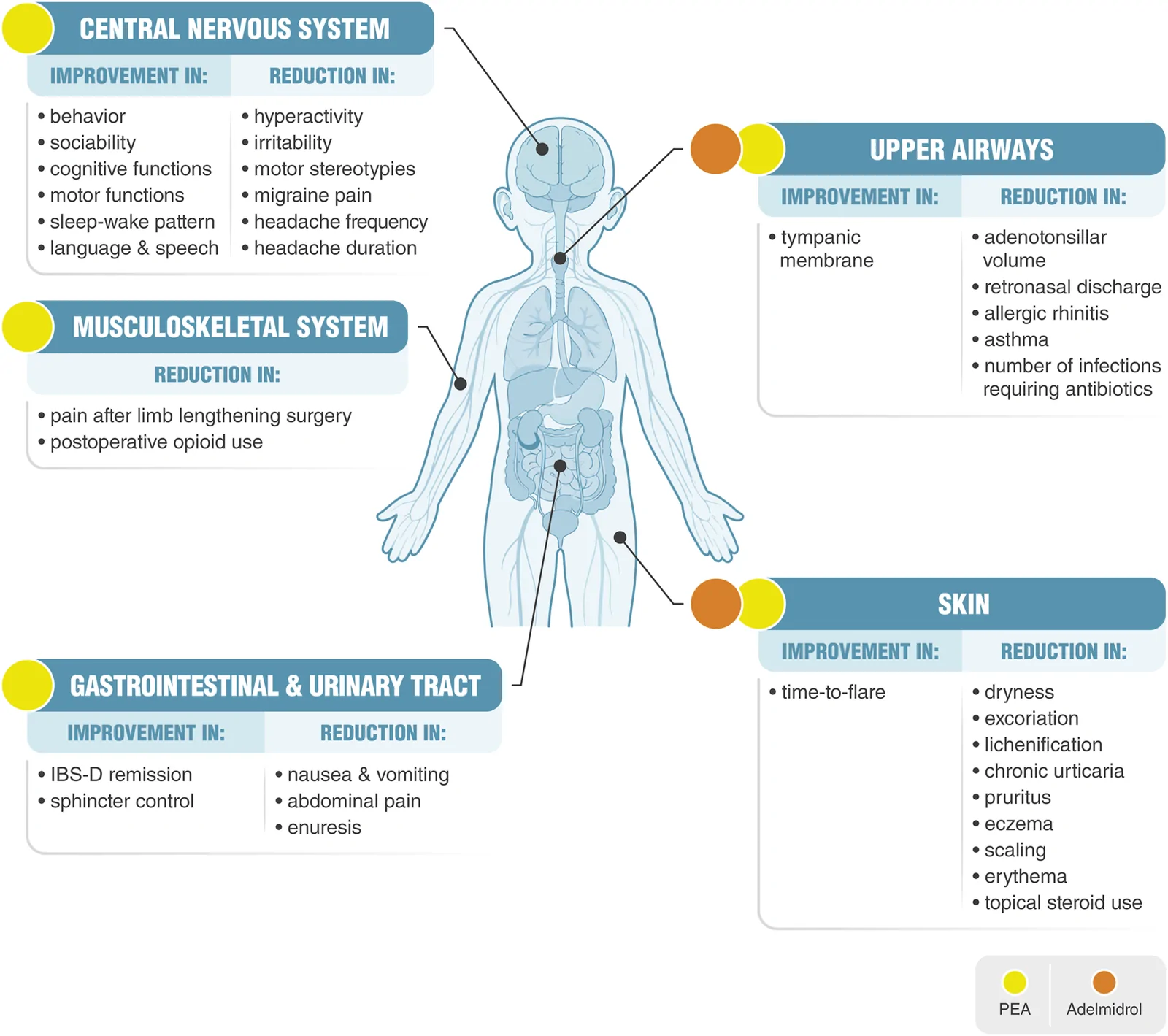
Schematic representation of the ALIA mechanism.

**TABLE 1:** Summary of the studies included in the review, organized by level of evidence (Oxford CEBM framework) and in reverse chronological order.

Study (Year)	Study design and LoE (oxford CEBM)	Clinical condition	Patients (n, gender)	Age (years, mean ± SD or range)	Formulation and dose	Treatment duration and regimen	Outcomes assessed	Key findings
Level 2	Randomized controlled trials	​						
Della Volpe et al., 2025	Randomized, pilot case-control study level 2	URTI	54 • 20 control (35% M) • 14 nasal SP (42.9% M) • 20 nasal + pharyngeal SP (50% M)	• 4.3 ± 1.2 (control) • 3.6 ± 1.1 (nasal SP) • 3.8 ± 1 (nasal + pharyng. SP)	Adelmidrol 2% nasal and/or pharyng. SP, TID	3 months	• Tympanic membrane, nose and pharynx state • Safety	• Superior airway and tympanic outcomes with combined nasal + pharyngeal SP followed by nasal SP alone, and then standard therapy • No adverse events
Di Nardo et al., 2024b	Multicenter, randomized, double-blind, placebo-controlled, parallel-arm trial level 2	IBS	70 • 34 co-micro PEAPol (47.1% M) • 36 Plb (44.4% M)	13.7 ± 2.20	Co-micro PEAPol 200 + 20 mg, TID	3 months	• IBS severity • Abdominal pain frequency and severity • Stools consistency • Bowel habits • Safety	• Higher remission rates and significant reduction in abdominal pain frequency in the co-micro PEAPol group • Greater reduction in IBS-SSS, pain intensity and life interference in the co-micro PEAPol group • No rescue medication use • No adverse events
Ameli and Ciprandi, 2019	Randomized, open-label, parallel-group trial level 2	RRI and house dust mite allergy	40 (45% M) • 20 PEA • 20 std therapy	6.5 (4–9)	PEA, bovine colostrum, phenylalanine, and probiotics combination (PEA 150 mg, QD*)	20 days/month for 3 months as add-on to std therapy (cetirizine drops 5 mg QD and intranasal budesonide 50 mcg per nostril QD)	• RRI and AOM number • Inferior and middle turbinate edema • Parents’ perception of treatment effectiveness • Sleep quality • Tolerability	• Greater reduction in the number of RRI and inferior turbinate edema in the PEA group vs. standard therapy • Improvement in AOM and sleep quality in both groups
Khalaj et al., 2018	Randomized, parallel-group, double-blind, placebo-controlled trial level 2	ASD	70 (62 completed) • 31 PEA (71% M) • 31 Plb (81% M)	• 6.8 ± 2.1 (PEA) • 7.4 ± 2.4 (Plb)	PEA 600 mg, BID	10 weeks as add-on to Rsp	• ASD-related behaviors • Safety	• Higher efficacy of PEA plus Rsp on irritability, hyperactivity, and inappropriate speech • No serious side effects
Del Rosso, 2007	Randomized, investigator-blinded, split-body trial level 2	AD	25 (56% M) • 25 PEA cream • 25 moisturizer	8 (4–15)	PEA cream, BID	Add-on to topical corticosteroid in acute phase (up to 4 weeks), followed by maintenance (12 weeks)	• Time-to-flare • Product preference • Safety	• Faster disease clearance during acute flare and prolonged time-to-flare during maintenance with PEA cream vs. moisturizer • Patient preference for PEA cream • No serious adverse events (transient stinging in 2 children on PEA cream)
Plesník et al., 1977	Randomized, double-blind, placebo-controlled trial level 2	RI	393 • 169 PEA • 224 Plb	11–15	PEA 46.5–74.2 mg/kg, QD	10 days (observation period: 8 weeks)	• Number of acute RI • Number of school absence days • Laboratory tests	• Greater reduction in acute RI and sick days in the PEA group • Greater effect of PEA in younger children
Rasková et al., 1974	Randomized, double-blind, placebo-controlled trial level 2	RI	466 (divided into two experimental groups): • Group 1 (33 PEA +34 Plb) • Group 2 (198 PEA +201 Plb)	1–6	PEA 50 mg/kg, QD (regimens from 12 days to 8 weeks)	• Group 1: 12 days (observation period: 8 weeks) • Group 2: 8 weeks	• Respiratory signs (rhinitis, cough, fever) • Sore throat • Otitis	• Greater reduction in respiratory infection incidence in the PEA group • No adverse events
Level 3	Controlled pilot or observational studies	​						
Nigro et al., 2014	Retrospective, controlled, observational study level 3	RRI	167 • 67 (PEA) • 100 (bacterial extracts)	• 4.5 ± 1.2 (PEA) • 4.7 ± 1.2 (bacterial extracts)	PEA, bovine colostrum, phenylalanine, and probiotics combination (PEA 150 mg, QD*)	20 consec. Days during the first month; 10 consec. Days during months 2–4	• Frequency of RI episodes requiring antibiotics	• Higher proportion of patients free from antibiotic-requiring infections • ≤ 2 RRI episodes in the PEA group
Level 4	Uncontrolled pilot or observational studies	​						
Kotzeva et al., 2025	Observational study (conference abstract) level 4	LE surgery-related pain	42 • 12 PEA • 30 control	13.5 (10-17) in the PEA group	umPEA 500 mg, QD	3 months as add-on to std analgesia§	• Pain intensity • Rescue drug use • Safety	• Greater reduction in pain and opioid use in the umPEA group • No adverse events
Bove, 2024	Preliminary observational study reported as a letter level 4	ASD	186	20 months–11 years	umPEA 600 mg, QD (10–15 days) followed by 600 mg, BID	6 months as add-on to behavioral/nutritional interventions	• ASD-related symptoms	• Improvement in language, communication, social interaction, attention, sleep-wake rhythm, psychomotor agitation and behavior
Papetti et al., 2020	Pilot open-label study level 4	Episodic migraine without aura	69 (26% M)	10.43 ± 2.8	umPEA 600 mg, QD	3 months as add-on to rescue medication (i.e., NSAIDs) for acute headache	• Monthly attack frequency • Attack intensity • Number of rescue drugs	• Reduction in attack frequency and intensity • Reduced monthly use of rescue medication • Fewer patients experiencing severe attacks • Mild side effects in only one patient
Eberlein et al., 2008	Multicenter, prospective observational, non-controlled study level 4	AD	923	5.5 ± 3.3	0.3% PEA cream, BID	4–6 weeks (as add-on to usual care°)	• AD symptoms • Itching intensity • Corticosteroid use	• Improvement in skin symptoms and itching intensity • Discontinuation of topical steroids in the majority of children
Pulvirenti et al., 2007	Pilot uncontrolled study level 4	AD	20 (55% M)	8 (3–16)	Adelmidrol 2% emulsion	4 weeks	• AD symptoms • Safety	• 80% complete resolution • No relapses at the 8-week follow-up • No improvement in untreated lesions • No adverse events
Level 5	Case reports	​						
Turrini et al., 2025	Case report (conference abstract) level 5	Neurodevelopmental disorders and severe headache	1 F	13	umPEA 5 mL (600 mg), QD#	4 treatment cycles§§ as add-on to ibuprofen as needed	• Headache frequency • Safety	• Reduction in headache frequency and duration • Reduction in headache-associated nausea/vomiting • Improved response to ibuprofen therapy • No adverse events
De Palma et al., 2018	Case report level 5	TBI	1 M	12	Co-ultra PEALut 700 + 70 mg, BID	10 weeks oral suspension, 6 weeks microgranules (as add-on to neurorehabilitation)	• Disability • Consciousness and cognitive function	• Progressive restoration of motor and cognitive functions • Improvement in bladder and bowel control
Bertolino et al., 2017	Case report level 5	ASD	1 M	10	Co-ultra PEALut 700 + 70 mg, BID	1 year in addition to gluten/casein-free diet	• ASD-related symptoms	• Improvement in behavior, hyperactivity, motor stereotypies and cognition • Reduction in enuresis
Antonucci et al., 2015	Case report level 5	ASD	2 M	13 and 15	um-PEA 300 mg, BID (1 week) followed by 600 mg, BID	1 or 3 months	• ASD-related symptoms • Atopic and respiratory symptoms • Safety	• Improvement in cognitive-behavioral and language outcomes • Reduction in atopic and respiratory symptoms • No adverse events

AD: atopic dermatitis; AOM: acute otitis media; ASD: autism spectrum disorder; BID: bis in die, twice daily; Co-microPEAPol: co-micronized PEA, and polydatin; Co-ultraPEALut: co-ultramicronized PEA, and luteolin; F: female; IBS: irritable bowel syndrome; IBS-SSS: IBS, severity scoring system; LE: limb elongation; LoE: level of evidence; M: males; NSAIDs: nonsteroidal anti-inflammatory drugs; Plb: placebo; QD: quaque die, once daily; RI: respiratory infections; RRI: recurrent respiratory infections; Rsp: risperidone; SP: spray; TID: ter in die, three times daily; TBI: traumatic brain injury; umPEA: ultramicronized PEA; URTI: upper respiratory tract inflammation.

*The dose of PEA, per sachet was derived from publicly available online sources.

#The correspondence between the administered volume of oral suspension (5 mL) and PEA, dose (600 mg) is reported on the manufacturer’s website.

§Paracetamol, NSAIDs, delorazepam and tramadol (on an as-needed basis).

°Topical corticosteroids, topical immune modulators, systemic antihistamines.

§§cycle 1: 16 months; cycles 2-3: 5 months each; cycle 4: 4 months.

A total of 2,530 children were evaluated, of whom 1,772 received PEA and 54 were treated with Adelmidrol administered topically. Among the studies investing oral treatments, eight used PEA in micron-sized formulations (either ultramicronized or co-(ultra)micronized with antioxidant polyphenols) (Antonucci et al., 2015; Bertolino et al., 2017; De Palma et al., 2018; Papetti et al., 2020; Bove, 2024; Di Nardo et al., 2024b; Kotzeva et al., 2025; Turrini et al., 2025), two employed a combination product containing PEA, bovine colostrum, phenylalanine and the probiotic Kluyveromyces B0399 (Nigro et al., 2014; Ameli and Ciprandi, 2019), and three used PEA in unspecified formulations (Rasková et al., 1974; Plesník et al., 1977; Khalaj et al., 2018). Regarding topical interventions, two studies investigated PEA-containing creams (Del Rosso, 2007; Eberlein et al., 2008), one evaluated an Adelmidrol emulsion (Pulvirenti et al., 2007), and one examined an Adelmidrol-based nasal spray, used alone or in combination with a pharyngeal spray containing Adelmidrol (Della Volpe et al., 2025).

### 3.2 Methodological quality and risk of bias assessment

In accordance with the SANRA guidelines and to complement the systematic approach inspired by PRISMA, a formal appraisal of the methodological quality and risk of bias was performed to transparently stratify the robustness of the available evidence across the different study designs.

The methodological reliability of the included randomized controlled trials (RCTs) evaluated via the Cochrane RoB 2 tool demonstrated an overall acceptable compliance with modern reporting guidelines, despite expected limitations linked to study specificities (Figure 3A). Across specific domains, the majority of the trials (Della Volpe et al., 2025; Di Nardo et al., 2024b; Ameli and Ciprandi, 2019; Khalaj et al., 2018; Del Rosso, 2007; Rasková et al., 1974) exhibited a highly robust profile, presenting a low risk of bias or only minor concerns regarding deviations from intended interventions (D2), missing outcome data (D3), and outcome measurement (D4). While six of these seven RCTs were judged as presenting “some concerns” from an overall perspective, this was primarily driven by the lack of pre-specified trial analysis plans—a common methodological standard missing in historical literature or trials conducted before modern registration registries became mandatory. Regarding the randomization process (D1), the vast majority performed adequately; the historical trial by Plesník et al. (1977) was rated at high risk of bias due to an alphabetical treatment allocation sequence. Notably, such pseudo-randomization represents a standard logistical practice of its era which does not invalidate the epidemiological baseline safety of the cohort, but simply reflects historical rather than contemporary procedural criteria.

**FIGURE 3:**
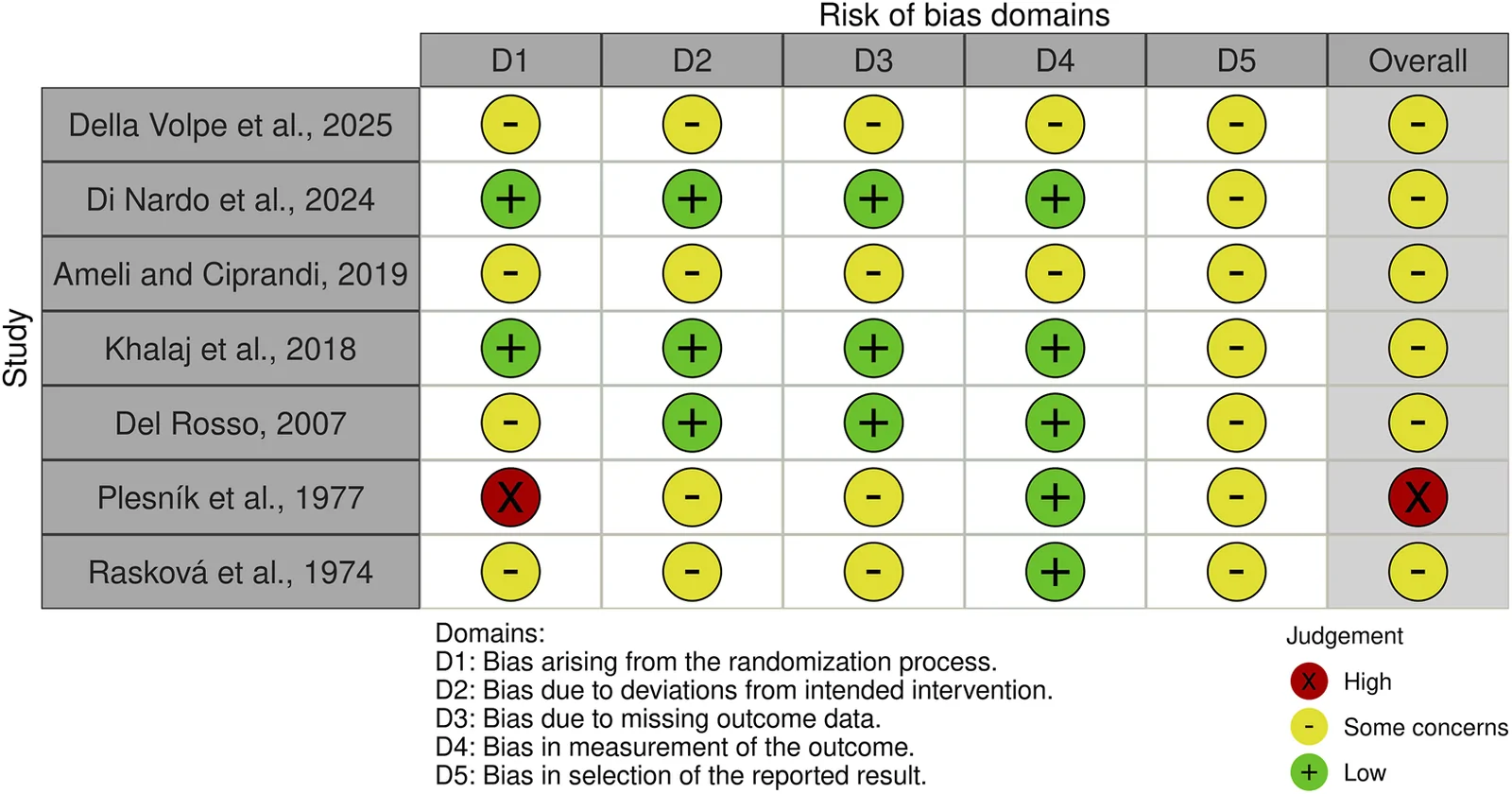
Risk of bias traffic light plots for randomized controlled trials evaluated using the Cochrane RoB 2.0 tool **(A)** and for non-randomized trials assessed using the Risk Of Bias In Non-randomized Studies–of Interventions (ROBINS-I) tool **(B)**.

For non-randomized studies appraised through the ROBINS-I framework, the evaluation highlighted a generally solid structure across most key domains, with low to moderate risks identified in the classification of interventions, deviations from intended designs, and missing data (Figure 3B). Outcome measurement (D6) was particularly robust, with only one study presenting a serious risk due to subjective endpoints (Papetti et al., 2020). The baseline judgment of serious risk of bias attributed to all five non-randomized studies (Bove, 2024; Papetti et al., 2020; Nigro et al., 2014; Eberlein et al., 2008; Pulvirenti et al., 2007) stemmed almost entirely from the potential influence of confounding (D1). This judgment reflects the inherent susceptibility of non-randomized and uncontrolled designs to baseline confounding, a well-documented limitation when evaluating observational data in pediatric clinical research.

Finally, the descriptive evidence provided by case reports demonstrated a very high level of methodological quality. According to the JBI critical appraisal checklist, all four clinical cases described across three publications (De Palma et al., 2018; Bertolino et al., 2017; Antonucci et al., 2015 which includes two patients) successfully fulfilled all eight appraisal criteria. This confirms a comprehensive and flawless reporting of patient traits, clinical history, interventions, outcomes, follow-up, and adverse events, ensuring optimal transparency.

Conversely, the two conference abstracts (Kotzeva et al., 2025; Turrini et al., 2025) were not formally appraised; the inherently restricted methodological and clinical details available in abstract formats precluded a reliable quality assessment via validated frameworks, as standardly accepted in rigorous literature syntheses.

### 3.3 Effects of PEA on neurological and neurocognitive symptoms in pediatric patients

In children, PEA has demonstrated beneficial effects on neurocognitive and neurological functions. In a 13-year-old boy with ASD, 1 month of supplementation with ultramicronized PEA (umPEA) led to marked behavioral improvements–including reduced tantrums, outbursts, self-talking and stereotypies–and enhanced expressive language both at home and at school, with an estimated two-year gain in speech level (Antonucci et al., 2015). Similarly, a 15-year-old male with ASD who had previously begun dietary restrictions and multivitamin supplementation, experienced further improvements in speech, sociability, cognitive functions, overall health, and behavior after 3 months of umPEA supplementation, with more modest gains in language (Antonucci et al., 2015). Bertolino and colleagues reported the outcomes of a 10-year-old male with ASD who had shown no benefit from psychotherapy, limited improvements from psychomotor and speech therapy, and mixed effects from dietary interventions (i.e., gluten/casein-free diet and vitamin supplements), which improved sleep–wake behavior and eye contact but worsened hyperactivity and stereotypies, ultimately necessitating suspension of the vitamins. Administration of PEA co-ultramicronized with luteolin (co-ultraPEALut) improved sociability, hyperactivity, motor stereotypies and cognitive function, making the child more affectionate, better able to follow simple commands, and capable of maintaining eye contact (Bertolino et al., 2017). In a preliminary observational study, children with full-blown, suspected, or subthreshold ASD receiving umPEA in combination with adjunctive interventions–such as melatonin, tryptophan, probiotics (lactobacilli and bifidobacteria), and fatty acids–were reported to show improvements in language, communication, and social interaction, as well as in attention, concentration, and sleep-wake patterns, alongside reductions in psychomotor agitation and behavioral problems (Bove, 2024). In a double-blind, placebo-controlled RCT, children with ASD and moderate to severe irritability demonstrated greater reductions in irritability and hyperactivity, as well as higher rates of appropriate speech when treated with risperidone plus PEA compared to risperidone plus placebo (Khalaj et al., 2018). Beyond ASD, in a 12-year-old boy with TBI in a minimally conscious state, 4 months of co-ultraPEALut supplementation combined with intensive neurorehabilitation progressively restored motor and cognitive functions (De Palma et al., 2018). Improvements began with eyelid closure communication and exploratory movements of the right hand, followed by enhanced head and torso stability and the ability to eat and drink in sips. The child subsequently developed well-organized communication skills and assisted walking. Finally, he achieved independence in basic self-care activities, including washing his face, eating, and brushing his teeth, surpassing expectations based on the authors’ experience with patients in similar initial conditions (De Palma et al., 2018). Finally, in allergic children with recurrent respiratory infections (RRI) treated with standard therapy alone or combined with a product containing PEA, bovine colostrum, phenylalanine and the probiotic Kluyveromyces B0399, sleep quality significantly improved in both groups, with no differences observed between them (Ameli and Ciprandi, 2019). Further details of the studies are presented in Table 1.

### 3.4 Effects of PEA on enuresis and gastrointestinal symptoms in children

Several case reports and clinical studies have highlighted improvements in autonomic and gastrointestinal function following umPEA or co-ultraPEALut supplementation, as summarized in Table 1. Bertolino et al. described a marked reduction of enuresis in a boy with ASD over the age of five after 14 months of co-ultraPEALut administration (Bertolino et al., 2017). Similarly, sphincter control improved in a 12-year-old boy with TBI. Whereas he initially required full assistance for bladder and bowel management up to 2 months post-injury, only moderate support was needed after 16 weeks of co-ultraPEALut supplementation (De Palma et al., 2018). In a 13-year-old girl with multiple neurodevelopmental disorders, umPEA was associated with decreased nausea and vomiting and improved daily functioning (Turrini et al., 2025). Complementing these findings, a recent RCT in children with IBS demonstrated that co-micronized PEA and polydatin (co-microPEAPol) significantly increased the proportion of patients achieving complete remission of gastrointestinal symptoms compared with placebo, particularly in those with diarrhea-predominant IBS, along with reduced interference of bowel issues with daily life (Di Nardo et al., 2024b).

### 3.5 Effects of PEA on children’s pain

Supplementation with umPEA has demonstrated efficacy in reducing pain in pediatric populations. In children with episodic migraine without aura, umPEA reduced attack frequency and intensity and decreased the monthly use of rescue medications (Papetti et al., 2020). Notably, clinical response was strongly dependent on treatment duration, with better outcomes observed in children receiving at least 12 weeks of supplementation compared to those treated for shorter periods (Papetti et al., 2020). In a 13-year-old girl with multiple neurodevelopmental disorders and severe headaches unresponsive to rescue drugs, umPEA reduced headache frequency and duration, improved response to ibuprofen, and mitigated the use of rescue medications, with symptoms worsening upon treatment discontinuation and improving upon reintroduction (Turrini et al., 2025). In pediatric IBS, co-microPEAPol significantly reduced abdominal pain frequency and intensity (Di Nardo et al., 2024b). Preliminary data from children undergoing limb elongation surgery indicated that 3 months of umPEA, in addition to paracetamol, nonsteroidal anti-inflammatory drugs (NSAIDs), delorazepam, and, if necessary, tramadol, reduced pain intensity and opioid requirements compared with standard care alone (NSAIDs, delorazepam, oxycodone) (Kotzeva et al., 2025). Additional information on the studies is provided in Table 1.

### 3.6 Effects of PEA and adelmidrol on respiratory, nasopharyngeal, and otologic symptoms

ALIAmides (i.e., PEA and Adelmidrol) have demonstrated beneficial effects on a range of respiratory and nasopharyngeal symptoms in pediatric populations. In children with RRI, both retrospective and placebo-controlled studies reported that PEA supplementation reduced the number of infections requiring antibiotics and increased the proportion of patients remaining free from clinically significant episodes, with particularly notable benefits in younger children (Rasková et al., 1974; Plesník et al., 1977; Nigro et al., 2014). When combined with standard therapy–cetirizine drops and intranasal budesonide–in children with RRI and house dust mite allergy, PEA further improved clinical outcomes, resulting in fewer RRI episodes, reduced edema of the inferior turbinate, and a significant decrease in the size of the middle turbinate compared with standard therapy alone, while no differences were observed in acute otitis media episodes, although reductions occurred in both groups (Ameli and Ciprandi, 2019).

Ultramicronized PEA used as a single treatment also produced clinically relevant improvements in a 13-year-old boy with ASD, asthma, allergic rhinitis, and allergic stigmata, whose previous pharmacologic treatments had been only temporarily effective or ineffective. After 1 month of umPEA supplementation, the patient showed reductions in nose-picking, asthmatic cough, nasal edema, and allergic stigmata (Antonucci et al., 2015). Upper respiratory tract inflammation was additionally investigated in children with adenotonsillar hypertrophy and recurrent otitis media, where treatment with Adelmidrol administered as both nasal and pharyngeal sprays yielded the greatest improvements in nasal, pharyngeal, and tympanic outcomes, followed by nasal spray alone and then standard therapy with mucolytics and steroids (Della Volpe et al., 2025). These findings indicate that ALIAmides exert effects across a cluster of pediatric symptoms involving the respiratory tract, nasopharynx, and middle ear, supporting their potential utility as adjunctive or standalone interventions for conditions that share overlapping inflammatory or immune-mediated mechanisms. Study-specific data are outlined in Table 1.

### 3.7 Effects of PEA and adelmidrol on dermatological symptoms

Topical ALIAmide formulations have demonstrated beneficial effects across several dermatological symptoms in pediatric populations. In children with mild to moderate atopic eczema, a multicenter, prospective, observational study showed that a PEA-containing cream improved dryness, excoriation, lichenification, scaling, erythema and pruritus, with itching rated as “very much better” compared with conventional creams. By the end of the study, most children had discontinued topical corticosteroids (Eberlein et al., 2008). In another study involving children with moderate symmetric atopic dermatitis, treatment was initiated at flare onset. One side of the body received a PEA cream combined with a mid-potency corticosteroid, while the other side was treated with a moisturizer plus the same corticosteroid. During the acute flare, faster clearance was observed on the PEA-treated side. During maintenance therapy, continued use of PEA prolonged the time to flare compared with the moisturizer, and most patients preferred the PEA cream (Del Rosso, 2007). One month of umPEA supplementation also provided dermatological benefits in a 13-year-old boy with ASD, reducing chronic urticaria and eczema that had previously responded only temporarily to treatment since early childhood (Antonucci et al., 2015). Topical Adelmidrol has shown clinical effects comparable to those of PEA. In children with mild atopic dermatitis, a four-week course of an Adelmidrol emulsion reduced erythema and pruritus, with complete resolution in most patients and no relapses at 8-week follow-up, whereas untreated lesions did not improve (Pulvirenti et al., 2007). Detailed information on each study is presented in Table 1.

### 3.8 Safety, tolerability and impact on rescue medication use of ALIAmides in pediatric populations

Both ALIAmides (PEA and Adelmidrol) have generally demonstrated favorable safety and tolerability profiles in pediatric populations, whether administered alone or with standard therapies. Oral PEA has been consistently shown to be safe since initial studies (Rasková et al., 1974; Plesník et al., 1977). Micron-sized formulations were well tolerated, with no significant adverse effects reported (Antonucci et al., 2015; Bertolino et al., 2017; De Palma et al., 2018; Bove, 2024; Di Nardo et al., 2024b; Kotzeva et al., 2025; Turrini et al., 2025), except for a single case of nausea and floating sensation (Papetti et al., 2020). Early evidence also indicates good tolerability in children younger than 3 years and in those receiving long-term supplementation (Bertolino et al., 2017; Bove, 2024; Turrini et al., 2025). Minor side effects, including headache and increased appetite, were observed with an undeclared PEA formulation, occurring at similar rates in children treated with risperidone plus PEA or risperidone plus placebo (Khalaj et al., 2018). The combination of PEA, bovine colostrum, phenylalanine, and probiotic was similarly well tolerated (Nigro et al., 2014; Ameli and Ciprandi, 2019). Topical PEA caused only transient stinging in a few cases, without leading to study discontinuation (Del Rosso, 2007). Adelmidrol delivered as an emulsion, pharyngeal spray, or nasal spray, produced no adverse effects in treated children (Pulvirenti et al., 2007; Della Volpe et al., 2025). Beyond safety, ALIAmides appear to exert clinically relevant effects across diverse pediatric conditions. Oral PEA in micron-sized formulations reduced or eliminated the need for rescue medications, respectively in children with migraine and IBS, and improved responsiveness to ibuprofen in a patient with severe neurodevelopmental disorders (Papetti et al., 2020; Di Nardo et al., 2024b; Turrini et al., 2025). In surgical settings, oral umPEA minimized the requirement for potent opioids compared with controls (Kotzeva et al., 2025). Topical PEA also allowed discontinuation of corticosteroid creams in the majority of treated children (Eberlein et al., 2008).

## 4 Discussion

The need for safe and effective non-pharmacological interventions is particularly pressing in pediatric care, where long-term pharmacological treatments are often limited by tolerability concerns, developmental considerations, and polytherapy burden. Within this framework, the present review provides a transdiagnostic synthesis of clinical evidence supporting the use of ALIAmides–namely, PEA, primarily administered orally, and its congener Adelmidrol, used topically–across a range of pediatric conditions (Figure 4).

**FIGURE 4:**
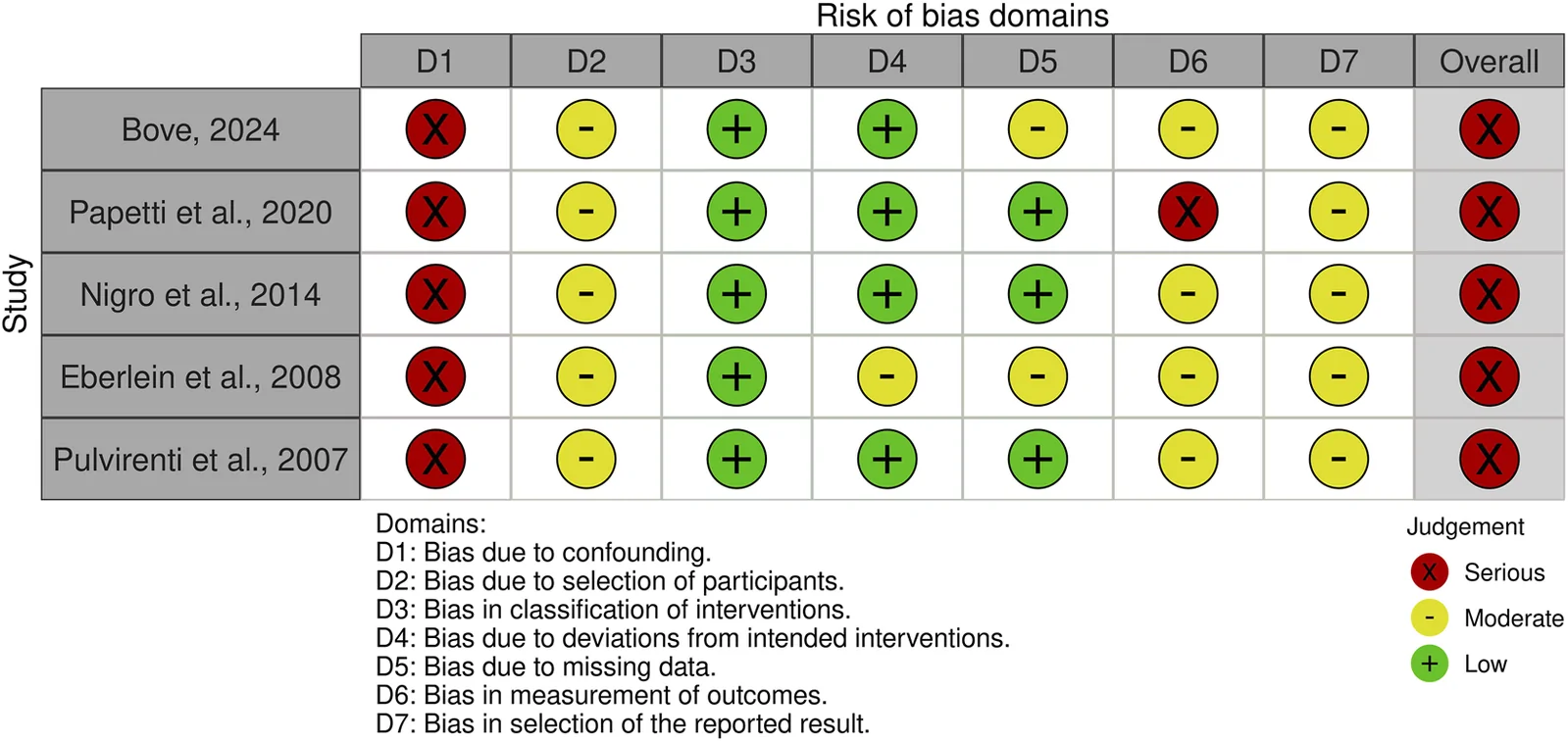
Clinical benefits of ALIAmides across organ systems involved in different pediatric conditions.

Their clinical effects may well be interpreted within a broader category of non-pharmacological interventions targeting shared biological processes. In particular, a unifying element emerging from both preclinical and clinical evidence is the central role of mast cells as key regulators at the interface between immune, nervous, and epithelial systems. Mast cells are present in almost all tissues, and are particularly notable in areas such as the mucosa in the respiratory, digestive, and urogenital systems, the dermis, lymph vessels, and in the proximity of peripheral nerves, allowing them to function as sensors of changes in their local microenvironment (Dileepan et al., 2023). Mast cells are increasingly recognized not only as effector cells in allergic and inflammatory responses, but also as active participants in neurodevelopmental processes (Chia et al., 2023). Dysregulation of mast cell activity has been hypothesized to influence neurodevelopmental trajectories, potentially contributing to conditions such as ASD, as well as to altered pain processing (Plum et al., 2024), gastrointestinal dysfunction (Ravanbakhsh and Kesavan, 2019), and respiratory reactivity (Wang et al., 2025). This broader perspective provides a biologically plausible framework linking apparently heterogeneous pediatric disorders through shared mast cell–driven mechanisms.

Within this context, the clinical effects of ALIAmides can be coherently interpreted. PEA acts as an endogenous autacoid that down-modulates mast cell activation and degranulation, thereby limiting the release of pro-inflammatory and pro-nociceptive mediators (Di Stefano et al., 2025). At the same time, PEA exerts direct effects on central neuroinflammatory processes by modulating microglial and astrocytic activation (Toti et al., 2023; Micheli et al., 2024; Coluzzi et al., 2025). The bidirectional crosstalk between mast cells and glial cells represents a key axis in the regulation of neuroinflammation, which, when persistent, may lead to maladaptive responses underlying a wide range of pediatric conditions, including neurodevelopmental disorders, migraine, and functional gastrointestinal disorders (Hendriksen et al., 2017; Ng et al., 2018; Biscetti et al., 2023; Hey et al., 2023; Aitella et al., 2026; Katiraei et al., 2026).

Adelmidrol, although structurally related to PEA, appears to act on mast cells predominantly through indirect mechanisms. Its amphipathic properties favor tissue penetration in topical applications, while its antioxidant and radical-scavenging activities contribute to the preservation of key extracellular matrix components, and to the reduction of oxidative stress–induced mast cell degranulation (Cavallaro et al., 2024). Through these mechanisms, Adelmidrol may exert local pro-homeostatic effects, particularly in tissues with high mast cell density, such as the skin and respiratory mucosa.

When interpreting these findings across different clinical domains—ranging from neurobehavioral symptoms to gastrointestinal, respiratory, and dermatological disorders—a critical distinction must be maintained between direct clinical efficacy and underlying mechanistic models. The clinical data collected in this review provide direct evidence of symptomatic improvement, whereas the shared involvement of mast cell and microglial modulation (i.e., the ALIA pathway) remains a highly plausible mechanistic framework rather than a directly verified conclusion. While this neuroinflammatory hypothesis offers a coherent biological basis for a transdiagnostic approach, direct trial data within the included pediatric studies did not measure these cellular biomarkers. Therefore, while the cross-condition clinical benefits are encouraging, the precise cellular pathways driving these effects in pediatric patients remain speculative and require formal confirmation through future biomarker-driven clinical trials.

This transdiagnostic synthesis must be interpreted with caution, as the included studies enrolled pediatric patients with markedly different disorders that vary in pathophysiology, disease severity, and clinical manifestations, thereby limiting the generalizability of the findings across all pediatric conditions.

In addition, cross-study comparisons are inherently limited by substantial heterogeneity in the formulations and dosages employed across the literature. The included protocols span from non-micronized, unspecified PEA formulations to advanced micron-sized technologies (such as ultramicronized PEA or PEA co-micronized with luteolin or polydatin), alongside multi-ingredient combinations containing bovine colostrum and probiotics. Such diversity precludes a uniform assumption of equivalent therapeutic action. Furthermore, the presence of additional compounds in some formulations may have contributed to the observed clinical effects through additive or synergistic mechanisms. Consequently, the findings should be interpreted with caution, as the potential involvement of the accompanying components cannot be completely excluded.

Nevertheless, when evaluating differences among the preparations employed, a consistent signal emerges regarding the impact of formulation technology on clinical efficacy. Specifically, co-micronized and ultramicronized PEA formulations appear to drive the most robust clinical outcomes. This is biologically plausible, as these particle-size reduction technologies exhibit significantly enhanced bioavailability, enabling the molecule to effectively reach both peripheral tissues and central nervous system targets (Petrosino and Di Marzo, 2017; Di Stefano et al., 2025). Conversely, unspecified or non-micronized formulations with suboptimal bioavailability appear less effective, particularly for clinical outcomes that heavily rely on central homeostatic mechanisms, such as sleep restoration or core behavioral dysregulation (Khalaj et al., 2018; Ameli and Ciprandi, 2019).

Importantly, when compared with other non-pharmacological or nutritional interventions targeting similar symptom domains, ALIAmides appear to show a more consistent pattern of benefit. For instance, improvements in irritability, hyperactivity, and communication have been reported inconsistently in children with ASD receiving interventions such as omega-3 fatty acids, probiotics, or dietary modifications, whereas more robust effects have been described only with high-dose folinic acid, albeit in selected subgroups (Taylor et al., 2018; Talib et al., 2024; Kostenko et al., 2026). In contrast, PEA-based interventions have been associated with improvements across multiple behavioral domains (Antonucci et al., 2015; Bertolino et al., 2017; De Palma et al., 2018; Khalaj et al., 2018; Bove, 2024), suggesting a broader underlying mechanism of action.

Similarly, in pediatric migraine, compounds such as riboflavin or melatonin have demonstrated some efficacy in reducing attack frequency or intensity, but evidence remains limited and heterogeneous (Sangermani and Boncimino, 2017; Orr, 2018; Yamanaka et al., 2021). The consistent reduction in pain intensity and frequency observed with umPEA, together with decreased reliance on rescue medications (Papetti et al., 2020; Turrini et al., 2025), suggests a potentially relevant role in modulating pain-related neuroinflammatory pathways.

In children presenting with functional gastrointestinal symptoms related to IBS, dietary approaches–such as low-FODMAP diets–and interventions with fibers or probiotics have yielded variable results, often confined to specific manifestations such as abdominal pain (Chakraborty et al., 2023; Di Nardo et al., 2024a). By comparison, the broader improvements reported with PEA in micron-sized formulations across both pain perception and bowel function (De Palma et al., 2018; Di Nardo et al., 2024b; Turrini et al., 2025) may be consistent with an upstream modulation of mast cell–mediated dysregulation of the gut–brain axis.

A similar pattern applies to respiratory and dermatological signs and symptoms. While evidence for traditional interventions–such as vitamins, probiotics, and prebiotics–in preventing or managing recurrent respiratory infections remains inconsistent (Milani et al., 2024; Zhang et al., 2025), lactoferrin offers more promising and consistent effects, despite a limited number of studies (Pasinato et al., 2024). In this scenario, both PEA and Adelmidrol have emerged as effective therapeutic options (Rasková et al., 1974; Plesník et al., 1977; Nigro et al., 2014; Antonucci et al., 2015; Ameli and Ciprandi, 2019; Della Volpe et al., 2025), likely by down-modulating mucosal mast cell activity and local inflammatory cascades. Admittedly, two foundational respiratory studies date back nearly 50 years (Rasková et al., 1974; Plesník et al., 1977). While these historical trials establish a robust, methodologically sound safety baseline in large cohorts, their clinical relevance must be evaluated in light of modern pharmaceutical technology. Specifically, research has evolved from the original, non-micronized formulation (Impulsin®) to advanced, micronized delivery systems that optimize bioavailability.

This protective barrier effect extends to dermatology. Topical ALIAmide-based formulations demonstrate clinically significant improvements in urticaria and atopic eczema (Del Rosso, 2007; Pulvirenti et al., 2007; Eberlein et al., 2008; Antonucci et al., 2015). Notably, these formulations have been shown to reduce the need for corticosteroids (Eberlein et al., 2008) —a steroid-sparing effect of critical importance in pediatric care.

Overall, these cross-condition clinical signals provide a conceptual rationale for the hypothesis that ALIAmides may interface with shared neuroinflammatory and mast-cell-driven pathways. However, a clear distinction must be maintained between verified clinical outcomes and mechanistic extrapolation. While the modulation of mast cell activation and microglial signaling offers an appealing, biologically plausible explanatory model for these overlapping pediatric symptom profiles, it remains a theoretical framework, as none of the reviewed trials measured clinical biomarkers. Consequently, while these findings highlight a promising transdiagnostic framework for addressing clinical symptom clusters through ALIAmides, the precise upstream molecular mechanisms operating in these pediatric cohorts remain to be formally confirmed.

Within this framework, safety emerges as a critical consideration, particularly in pediatric populations and in clinical settings requiring long-term or repeated treatment courses. Preclinical toxicological evidence strongly supports the favorable safety profile of PEA. In a prenatal developmental toxicity study conducted in Wistar rats, oral administration of PEA at doses up to 1,000 mg/kg/day throughout gestation did not result in maternal toxicity, reproductive impairment, or fetal abnormalities. Under these experimental conditions, the no-observed-adverse-effect level (NOAEL) was established as greater than 1,000 mg/kg/day, corresponding to a human equivalent dose exceeding 9.7 g/day (Deshmukh et al., 2021). Notably, this safety threshold far exceeds the dosages employed in the pediatric populations considered in this review, which collectively provide an overview of the dosing regimens most frequently reported in the available literature. Among the conditions most extensively investigated, a daily dose of 600 mg of micron-sized PEA was the regimen most commonly employed for pain-related disorders, including migraine and IBS. In studies involving neurodevelopmental disorders, higher doses (typically 1,200 mg/day, administered in two divided doses) were more commonly used. Moreover, when umPEA is administered, the authors described a titration strategy (Antonucci et al., 2015; Bove, 2024), consisting of an initial dose of 600 mg/day for the first 10–15 days, followed by an increase to the full dose. However, given the inherent heterogeneity of treatment durations, formulations and clinical cohorts, definitive recommendations regarding optimal individual dosing strategies cannot be drawn at this stage.

The robust preclinical safety profile, along with the low doses used, is consistent with clinical observations in pediatric populations, where both PEA–particularly in micron-sized formulations–and Adelmidrol have generally demonstrated good tolerability (Rasková et al., 1974; Plesník et al., 1977; Pulvirenti et al., 2007; Nigro et al., 2014; Antonucci et al., 2015; Bertolino et al., 2017; De Palma et al., 2018; Ameli and Ciprandi, 2019; Bove, 2024; Di Nardo et al., 2024b; Della Volpe et al., 2025; Kotzeva et al., 2025; Turrini et al., 2025). Across the studies included in this review, no clinically relevant adverse effects were reported, even during prolonged administration and in very young children (Bertolino et al., 2017; Bove, 2024; Turrini et al., 2025).

Overall, this review suggests that PEA, particularly in its micron-sized formulations, and Adelmidrol may represent valuable options for the management of a range of pediatric conditions, given their generally favorable safety and tolerability profiles. This aspect is especially relevant in children with chronic disorders requiring long-term or multiple pharmacological treatments, where the inclusion of well-tolerated, non-pharmacological interventions may help reduce overall therapeutic burden.

Despite these encouraging findings, several limitations should be acknowledged. First, the included studies exhibit substantial methodological heterogeneity in terms of study designs, patient populations, sample sizes, and outcome measures, alongside methodological limitations identified through the quality and risk of bias assessments. Second, as highlighted above, direct cross-study comparisons are significantly constrained by the wide variability in pediatric conditions, PEA formulations, particle-size technologies (e.g., non-micronized, ultramicronized, or co-micronized), and dosages. Furthermore, the inclusion of single-case reports and small pilot trials warrants caution, as these findings may not be fully generalizable to broader pediatric and adolescent populations. This heterogeneity, along with the presence of multi-ingredient combinations, precludes a formal quantitative meta-analysis and limits our ability to establish uniform clinical guidelines at this stage.

Finally, although a favorable tolerability profile and no major safety concerns were reported across the included studies, the currently available evidence does not yet allow definitive conclusions regarding the long-term safety of PEA in pediatric populations.

Notwithstanding these limitations, the convergence of mechanistic plausibility with consistent clinical signals observed across diverse pediatric conditions provides a solid rationale for further investigation. Well-designed randomized controlled trials are warranted to confirm the clinical efficacy and safety of PEA and Adelmidrol, to optimize formulations and dosing strategies, and to better define their role within integrated, non-pharmacological, mechanism-based approaches to pediatric care.

## 5 Conclusion

Maintaining physiological homeostasis is fundamental to children’s health and development. Within this perspective, the identification of safe, well-tolerated, and physiologically oriented interventions capable of supporting endogenous regulation of immune-inflammatory processes is increasingly relevant, particularly for pediatric conditions characterized by chronicity, symptom overlap, and multifactorial pathophysiology.

However, given the substantial methodological and formulation heterogeneity among the included studies, along with the preliminary nature of the current evidence base, these findings must be interpreted with caution. Rather than supporting definitive clinical recommendations, the physiological, homeostatic mode of action of ALIAmides provides an encouraging rationale for further investigation through well-standardized, randomized controlled trials. Such future research is essential to rigorously evaluate the potential role of ALIAmides as supportive, transdiagnostic interventions in pediatric chronic conditions characterized by immune-inflammatory dysregulation.
